# An Information Gain-Based Model and an Attention-Based RNN for Wearable Human Activity Recognition

**DOI:** 10.3390/e23121635

**Published:** 2021-12-06

**Authors:** Leyuan Liu, Jian He, Keyan Ren, Jonathan Lungu, Yibin Hou, Ruihai Dong

**Affiliations:** 1 Faculty of Information Technology, Beijing University of Technology, Beijing 100124, China; chinaleyuan@emails.bjut.edu.cn (L.L.); JonathanLungu@emails.bjut.edu.cn (J.L.); ybhou@bjut.edu.cn (Y.H.); 2 Beijing Engineering Research Center for IOT Software and Systems, Beijing University of Technology, Beijing 100124, China; 3 School of Computer Science, University College Dublin, D04 V1W8 Dublin 4, Ireland; ruihai.dong@ucd.ie

**Keywords:** human activity recognition, information gain, attention mechanism, Attention-RNN

## Abstract

Wearable sensor-based HAR (human activity recognition) is a popular human activity perception method. However, due to the lack of a unified human activity model, the number and positions of sensors in the existing wearable HAR systems are not the same, which affects the promotion and application. In this paper, an information gain-based human activity model is established, and an attention-based recurrent neural network (namely Attention-RNN) for human activity recognition is designed. Besides, the attention-RNN, which combines bidirectional long short-term memory (BiLSTM) with attention mechanism, was tested on the UCI opportunity challenge dataset. Experiments prove that the proposed human activity model provides guidance for the deployment location of sensors and provides a basis for the selection of the number of sensors, which can reduce the number of sensors used to achieve the same classification effect. In addition, experiments show that the proposed Attention-RNN achieves F1 scores of 0.898 and 0.911 in the ML (Modes of Locomotion) task and GR (Gesture Recognition) task, respectively.

## 1. Introduction

Human activity recognition (HAR) technology [1] has been widely used in various areas, such as security monitoring [2], human-machine interaction [3], sports analysis [4], medical treatment [5], and health care [6], etc. According to the types of sensors used, HAR systems can be mainly divided into environmental sensor-based HAR, video-based HAR, and wearable sensor-based HAR [7]. However, environmental sensor-based HAR requires placing sensors in a fixed environment, which may cause certain limitations [8,9]. Although video-based HAR systems have made great progress, as the nature of this kind of system requires using cameras to collect human activities and record as videos for data analysis, this would raise several issues, such as susceptibility to light and occlusion, vulnerability of privacy protection, and large data processing volume [10]. Wearable sensor-based HAR systems integrate sensors, e.g., accelerometers, magnetometers, and gyroscopes, into wearable devices such as smartphones, bracelets, smart glasses, helmets, etc., and human body data is collected through these devices [11]. Wearable sensor-based HAR has become popular due to its convenience of application and ability to protect user privacy. Researchers have developed a variety of wearable sensor-based HAR solutions. For example, Fu et al. integrated multiple heterogeneous sensors into a wireless wearable sensor node for HAR and proved that the multi-modal data could achieve a better accuracy [12]. Iqbal et al. used smartphones to collect the data and transferred these collected data to a data server for processing and analysis [13].

The wearable sensor-based HAR can be divided into three stages: data perception, feature extraction, and activity classification. In the data perception stage, since wearable sensor-based HAR systems lack unified protocols and specifications, the types, numbers, and deployment locations of sensors in each system are different. For example, Köping et al. deployed eight inertial sensors into an HAR system, which consisted of a mobile phone, a glass, and a watch [14]. Hegde et al. combined insole-based and wrist-worn wearable sensors for HAR [15]. Davidson et al. integrated accelerometers, gyroscopes, compasses, barometers, and a GPS receiver into a device on the back of the body for analysis of running mechanics [16]. Due to the different types and deployment locations of wearable HAR sensors, it is difficult to popularize and apply the HAR algorithms. In the past, there have been a few studies on the number and location of sensors for wearable sensor-based HAR. Sztyler et al. used a classifier for location selection and analyzed the impact of 7 different sensor locations on the HAR results [17]. However, this method relied heavily on the accuracy of the classifier and only obtained the position of one sensor. Atallah et al. measured the importance of each location by calculating the overall weight of 13 artificial characteristics [18]. This method relied too much on the selection of features by manual experience. In recent years, some researchers have applied some methods based on information theory in their perception systems. For example, Jin et al. used causal entropy to select high causal measures as input data, but did not study the location of sensor deployment [19]. Lee et al. estimated the posture stability of the elderly through permutation entropy, but only used a sensor fixed on the back [20].

In the feature extraction stage and activity classification stage of wearable sensor-based HAR, technology development has gone through the traditional machine learning period and the current deep learning period. Traditional machine learning relies on artificial features, while deep learning can automatically extract features. Artificial features refer to the features artificially constructed by experts through in-depth analysis and enlightening thinking of the original data with the help of domain knowledge, which requires a lot of human resources. Traditionally, various classical machine learning algorithms [21], such as random forest [22], Bayesian network [23], Markov model [24], and support vector machine (SVM) [25], were used for analyzing wearable HAR data. In a strictly controlled environment, the traditional machine learning algorithms discussed can obtain excellent results. However, they need professional domain knowledge for manual feature extraction and complex preprocessing steps [26]. In recent years, deep learning algorithms have been applied to HAR and achieved outstanding performances. For instance, Ignatov used a CNN to automatically extract features from human activity data and combined them with artificial features to achieve relatively excellent results on the WISDM dataset and UCI-HAR dataset [27]. The limitation of Ignatov’s work is that artificial features were still necessary, i.e., its data processing was inefficient, as it still required professional domain knowledge. Ronao and Cho used mobile phone accelerometer data and gyroscope data to classify six human activities and achieved an overall accuracy of 95.75% [28]. Since only one mobile phone device was used, the range of perception was limited and only a few simple human activities could be recognized. Aiming to mine temporal and spatial characteristics of human activities, Ordóñez et al. proposed a deep neural network (namely DeepConvLSTM), which benefits from both LSTM and CNN architectures [29]. Its weighted F1 scores of the daily activity recognition task and the 18-class gesture recognition task on the UCI Opportunity Challenge dataset [30] reached 0.895 and 0.915, which was significantly higher than the pure CNN. Vaswani et al. used the attention mechanism for machine translation task and achieved excellent results [31]. Then the attention mechanism can also be applied in HAR. Although the deep learning algorithms work well in HAR, their complex structures require high computing and storage resources, and require special processor support, such as GPU, to meet the needs of real-time HAR.

Aiming at the problems of lacking unified standards for sensor placement and the over-complexity of deep learning classification algorithms in the current wearable sensor-based HAR, this paper proposes a new HAR method. First, an information gain-based human activity model is established according to the characteristics of the human skeleton structure. It serves as a standard for the placement location and number of sensors in the perception stage. Second, a deep neural network (namely Attention-RNN) combined with the attention mechanism and bidirectional LSTM (BiLSTM) is designed to extract the features of human activity data and classify the data. Finally, on the public UCI Opportunity Challenge dataset, the balance effect of Attention-RNN in F1 score and running speed is verified, and the effect of the information gain-based human activity model is verified. The follow-up content of this paper is organized as follows: in Section 2, the information gain-based human activity model is presented. Section 3 elaborates on the architecture and principles of Attention-RNN. Section 4 introduces the UCI Opportunity Challenge dataset, the Attention-RNN training, the performance metrics, the experiments on Attention-RNN, and the experiments on information gain-based human activity model. Because experiments on the information gain-based human activity model need to use the Attention-RNN for effect evaluation, experiments are performed first to verify the effectiveness of Attention-RNN. Section 5 summarizes the entire text and prospects for the follow-up research directions.

## 2. Information Gain-Based Human Activity Model

In the process of human activities, different parts of the human body can exhibit different movement characteristics. The location and number of sensors are key issues in wearable sensor-based HAR. A large number of studies have discovered the positions to place sensors on the human body: head, ears, neck, torso, chest, abdomen, back, waist, pelvis, buttocks, hands, wrists, arms, feet, ankles, calves, thighs, knees, and so on. Yu et al. summarized these positions into the following categories: head, upper limbs, chest, waist back hip, lower limbs, and feet [32]. In 2010, Microsoft released Kinect, a device that can collect color images and depth images. The skeleton API in the Kinect for Windows SDK could provide position information of up to two people in front of Kinect, including detailed postures and 3D coordinate information of bone points. In addition, Kinect for Windows SDK could support up to 20 bone points. The data object type was provided as skeleton frames, and each frame could save up to 20 points [33]. Based on the past research and the human skeleton model proposed by Microsoft, this paper proposes the information gain-based human activity model.

**Figure 1:**
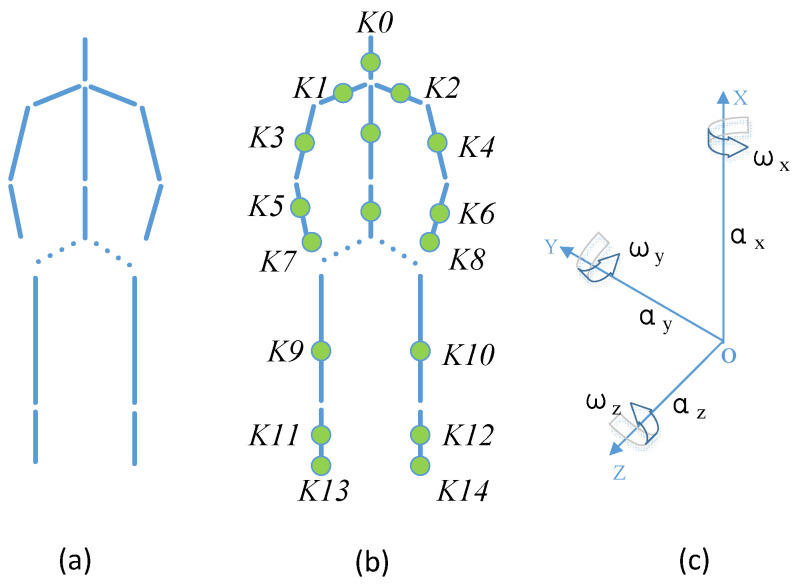
Information gain-based human activity model. (**a**) Human skeleton. (**b**) Positions of sensors can be fixed. (**c**) Cartesian coordinate system.

According to the relationship between bones and joints, bones can be regarded as rigid bodies, and joints can be regarded as connecting mechanisms [34]. Therefore, in the modeling of the articulated skeleton, the human body can be considered as a motion mechanism composed of multiple linkages and multiple joints. Figure 1 shows an example of the proposed human activity model. The skeleton of the model is composed of 15 linkages and 17 joints. Among them, 13 linkages are suitable for placing sensors, and the two linkages of the span are not suitable for placing sensors, which have been shown by dotted lines, as shown in Figure 1a. The deployable sensor nodes set of model is *P* = {*K*0, *K*1, *K*2,…, *K*14}, as shown in Figure 1b, where *K*0 is the head perception node, *K*1 and *K*2 are shoulder perception nodes, *K*3, *K*4, *K*5 and *K*6 are upper limb perception nodes, *K*7 and *K*8 are hand perception nodes, *K*9, *K*10, *K*11 and *K*12 are lower limb perception nodes, and *K*13 and *K*14 are foot perception nodes.

The joints of the proposed model all have three degrees of freedom, namely around the *X*-axis, around the *Y*-axis, and around the *Z*-axis. In order to standardize the expression of human activity, this paper adopts the spatial Cartesian rectangular coordinate system [35] to establish a unified human activity model. In Figure 1c, *a_x_*, *a_y_*, and *a_z_* represent the acceleration component data collected by the 3-axis accelerometer along the *X*-axis, *Y*-axis, and *Z*-axis in the coordinate system during human activities; *ω_x_*, *ω_y_*, and *ω_z_* represent the angular velocity component data of the human body sensed by gyroscope along *X*, *Y* and *Z* axes. Only the components of acceleration and angular velocity on each axis are shown in Figure 1c. In fact, each axis may contain other components, such as magnetic force. Suppose *A* is the human activity, *F_ext_* is the feature extraction function, and *F_cls_* is the human activity classification function, then the human activity can be expressed by Equation (1).

*A* = *F_cls_* (*F_ext_* (*K*0, *K*1, *K*2,…, *K*14)) (1)

note that:

(2) $$Ki=(a_{x}^{i},\;a_{y}^{i},\;a_{z}^{i},\;\omega_{x}^{i},\;\omega_{y}^{i},\;\omega_{z}^{i}\ldots )$$

The contribution of *Ki* to HAR is an important basis for sensor deployment. The human activity model uses information gain [36] to measure the degree of contribution. Information gain is an evaluation method based on entropy. It measures the contribution of feature *F* to the classification model. It is generally defined as the difference between the information entropy of all category *A* before and after the feature *F* appears, as shown in Formulas (3)–(5).

(3) InfoGain(F,A)=H(A)−H(A|F)

(4) $$H\left(A\right)=-\overset{m}{\underset{j=1}{\sum }}P\left(A_{j}\right)\log P\left(A_{j}\right)$$

(5) $$H\left(A|F\right)=-\underset{j}{\sum }\underset{v\in F}{\sum }P\left(A_{j}|F=v\right)\log P\left(A_{j}|F=v\right)$$

where *H*(*A*|*F*) and *H*(*A*) are respectively the information entropy when the feature *F* appears or not. The *v* in Equation (5) belongs to the set *F*, that is, v∈F. In addition, *P*(*A_j_*) is the prior distribution of category probabilities and *P*(*A_j_*|*F* = *v*) is the posterior probabilities.

The information gain of *Ki* is the sum of the information gain of all its channels, as shown in Formula (6). Among them, $\mathrm{InfoGain}\left(K_{il}\right)$ represents the information gain of *Ki*’s *l*th channel, and $C_{i}$ represents the total number of *Ki*’s sensor channels.

(6) $$\mathrm{InfoGain}\left(K_{i}\right)=\sum_{l=1}^{C_{i}}\mathrm{InfoGain}\left(K_{il}\right)$$

Then sort all sensor nodes according to the information gain value, and adopt the greedy strategy to select the optimal sensor combination with the top contribution. Human activity can finally be expressed by Equation (7). *K_top_i_* represents the sensor whose information gain ranks *i*.

*A* = *F_cls_* (*F_ext_* (*K_top_1_*, *K_top_2_*,…, *K_top_i_*,…)) (7)

## 3. Attention-RNN for Wearable HAR

**Figure 2:**
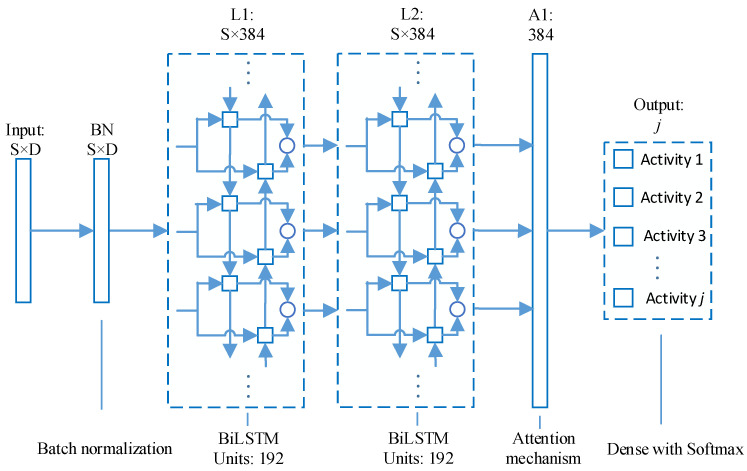
Network architecture of the Attention-RNN.

A deep learning network based on an attention mechanism, named Attention-RNN, is designed to realize wearable HAR. The architecture of Attention-RNN is shown in Figure 2, including 1 input layer, 1 batch normalization (BN) layer, 2 BiLSTM layers, 1 attention layer, 1 dense layer, and 1 output layer.

The first layer of Attention-RNN is the input layer. The input data (*X*_1_, *X*_2_, *X*_3_… *X_t_*… *X_n_*) is a matrix of *n* × S × D, where D is the number of sensor channels, and S is the number of temporal data for each sensor channel.

The second layer is a batch normalization (BN) layer. Ioffe and Szegedy’s research proved batch normalization method [37] could reduce the number of training steps required for model convergence, and could use a larger learning rate without paying too much attention to the initialization parameters and dropout. Therefore, a batch normalization layer is used here to simplify and speed up the training of the network.

The third layer (L1) and the fourth layer (L2) are both BiLSTM layers, and each layer has 192 units. The L1 layer outputs the sequence, which serves as the input of L2. Karpathy et al. proved through experiments that over two recurrent layers are more effective in predicting temporal events [38], so two BiLSTM layers are added after the BN layer. The Tanh function is used as the activation function when generating candidate memories. Because the output of the Tanh function is −1 to 1, which is consistent with the feature distribution of most scenes centered on 0, and the Tanh function has a larger gradient than the Sigmoid function near the input of 0, which can speed up the model convergence. L2 outputs the hidden state values of all time steps as the input to the next layer (A1). BiLSTM consists of forward LSTM and reverse LSTM. Each LSTM memory block is composed of a forget gate, an input gate, and a memory cell. The calculation process of BiLSTM is shown in Equations (8)–(16). In Equations (8)–(13), $x_{t}$ is the input information at the current moment, $f_{t}$ is the forgetting factor of the forgetting gate, $i_{t}$ is the output of the input gate, ${\tilde{C}}_{t}$ is the candidate value of the cell, $C_{t}$ is the cell state, $o_{t}$ is the output of the output gate, and $h_{t}$ is the output of the LSTM memory block. In Equations (14)–(16), $h_{f}$ and $h_{r}$ represent the output of forward LSTM and reverse LSTM, respectively. The output of BiLSTM is $H_{t}$. In addition, w and b in the equations are the corresponding weight coefficient matrix and bias term.

(8) $$f_{t}=\sigma \left(W_{f}\left[h_{\left(t-1\right)},x_{t}\right]+b_{f}\right)$$

(9) $$i_{t}=\sigma \left(W_{i}\left[h_{\left(t-1\right)},x_{t}\right]+b_{i}\right)$$

(10) $${\tilde{C}}_{t}=tanh\left(w_{c}*\left[h_{\left(t-1\right)},x_{t}\right]+b_{c}\right)$$

(11) $$C_{t}=f_{t}C_{\left(t-1\right)}+i_{t}*{\tilde{C}}_{t}$$

(12) $$o_{t}=\sigma \left(w_{o}*\left[h_{\left(t-1\right)},x_{t}\right]+b_{o}\right)$$

(13) $$h_{t}=o_{t}*tanh\left(C_{t}\right)$$

(14) $$h_{f}=f\left(w_{f1}x_{t}+w_{f2}h_{t-1}\right)$$

(15) $$h_{r}=f\left(w_{r1}x_{t}+w_{r2}h_{t+1}\right)$$

(16) $$H_{t}=g\left(w_{o1}*h_{f}+w_{o2}*h_{r}\right)$$

The A1 layer is an attention mechanism layer. The attention mechanism is designed according to the importance of the temporal characteristics of human activities at different moments, as shown in Equations (17)–(19). Among them, $u_{t}$ is the hidden layer unit,$\;a_{t}$ is the weight coefficient vector, $H_{t}$ is the output of BiLSTM, $v_{t}$ is the output vector of the attention mechanism, $w_{w}$ is the weight coefficient matrix from L2 to A1, and *b* is the bias. The vector $u_{w}$, which is randomly initialized and learned during training, is introduced to capture temporal context. The similarity, which is used as a measure of importance, is obtained by dot product $u_{t}$ and $u_{w}$. The normalized weight coefficient vector $a_{t}$ is obtained through the Softmax function. The time attention mechanism assigns different weights to the characteristics of human activities at different moments so that the characteristics at important moments receive more attention to improve the accuracy of HAR.

(17) $$u_{t}=tanh\left(w_{w}H_{t}+b\right)$$

(18) $$a_{t}=\mathrm{softmax}\left(u_{t}^{T}u_{w}\right)$$

(19) $$v_{t}=\sum a_{t}H_{t}$$

The last layer is a dense layer, which is also an output layer. The units of this layer are set to the number of human activity categories to be classified, which should be consistent with the number of label categories of the human activity dataset. Softmax is used as the activation function, as shown in Equation (20), where $v_{t}$ is the output vector of A1, $w_{j}$ is the weight matrix from A to the output layer, $b_{j}$ is the offset corresponding to $w_{j}$. Softmax maps the results of various classes to the probability between 0 and 1, and the class with the highest probability is the predicted class.

(20) $$y_{j}=\mathrm{softmax}\left(w_{j}v_{t}+b_{j}\right)$$

## 4. Experiments and Analysis

### 4.1. Dataset

**Figure 3:**
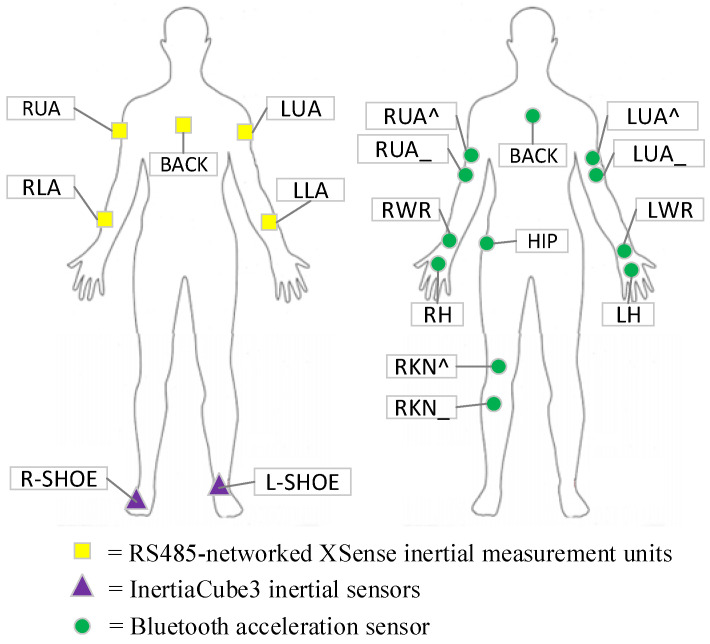
Sensors placement of the dataset.

The public UCI Opportunity Challenge dataset is used as the experimental dataset, which has 113 data channels (each sensor axis one channel). The dataset was recorded by 19 sensors fixed on the body of the subjects and the sampling frequency was 30 Hz. As shown in Figure 3, five yellow squares represent the RS485-networked XSense inertial measurement unit (IMU). Two purple triangles represent InertiaCube3 inertial sensors, and 12 green circles represent Bluetooth acceleration sensors. Each XSense IMU comprised a 3-axis accelerometer, a 3-axis gyroscope, and a 3-axis magnetometer. Each InertiaCube3 included a gyroscope, magnetometers, and accelerometer. The dataset recorded two types of activity data: Drill type, where subjects performed a set of pre-defined activities in sequence, and ADL (activity of daily life) type, where subjects performed high-level activities (getting up, grooming, preparing breakfast, cleaning). These high-level tasks included multiple atomic activities (for example, preparing breakfast includes preparing sandwiches, preparing coffee, drinking water, and other atomic activities), and there was no limit to the order in which atomic activities were performed. The dataset contains 1 Drill activity and 5 ADL activities of 4 subjects. In the Opportunity Challenge, task A and task B were to classify 5 Modes of Locomotion (ML) and recognize 18 gestures (GR) respectively. Since the data of subject 4 added noise in the challenge to perform other tasks, we only used the data of subjects 1, 2, and 3. The dataset was divided into the training set and testing set consistent with the Opportunity Challenge. The ADL4 and ADL5 of subjects 2 and 3 constituted the testing set. The remaining activities of subjects 1, 2, and 3 were used as the training set.

The linear interpolation method was used to fill the missing values of the dataset in the temporal direction. Since the records of the dataset were continuous, a sliding window with a length of 24 and a sliding step of 12 was used to segment the continuous records. The label of the last data in the sliding window was used as the label of the intercepted sample. The final intercepted dataset is shown in Table 1. The Null class in the table represents data that is not of interest.

### 4.2. Attention-RNN Training

All experiments were carried out on a server with the Ubuntu system. The GPU of the server was TITAN Xp 12G, and the CPU was Intel Xeon E5-2620 v4. The RAM size of the server was 62 G. The experiments program was coded in Python 3.7. Pandas [39] and Numpy [40] were used for data processing, and Keras [41] was used to realize the Attention-RNN network. The CuDNNLSTM in Keras was used to construct the network to improve the speed of the network.

During training, a random 5% of the training data was used to verify the loss and F1 at the end of each epoch. The Adadelta method [42] with adaptive learning rate was used as the network parameters optimizer. The initial learning rate of 1.0 and the batch size of 16 were used for network training. The early stopping mechanism was used to stop the training automatically. If the training loss did not decrease after 50 epochs, the training would be stopped, otherwise, the training would continue. The verification F1 was monitored, and only the model with the highest verification F1 rate was saved.

### 4.3. Performance Metrics

Due to the imbalance of the dataset in different classes, it is more reasonable to use the F1 score as the performance metric. The F1 score combines the effects of precision rate and recall rate, as shown in Equation (21):

(21) $$F_{1}=\sum F_{j}=\sum \frac{N_{j}}{N}\cdot \frac{2P_{j}\cdot R_{j}}{P_{j}+R_{j}}$$

where *j* is the class index, and $N_{j}$ is the number of samples of class *j*. *N* is the total number of samples. $P_{j}$ and $R_{j}$ are the precision rate and recall rate of class *j*, respectively.

The confusion matrix is suitable for visualizing the classification results of each class. The vertical axis of the confusion matrix is the actual class, and the horizontal axis is the predicted class. The sum of each column is the number of samples predicted as each class, and the sum of each row is the number of each class in the dataset. The background of each grid of the confusion matrix is filled with color according to the numerical value (the larger the numerical value, the darker the color).

### 4.4. Results and Discussion

#### 4.4.1. Experiments on Attention-RNN

Table 2 shows the F1 comparison between the proposed Attention-RNN and the classification techniques published in the past. In the ML task, the F1 score of the proposed Attention-RNN was 0.898, which was over 3% higher than Random Forest [43] and was 0.03 higher than the best DeepConvLSTM [29]. In the GR task, the F1 score of the proposed Attention-RNN was 0.911, which was higher than Random Forest and CNN [44], but slightly lower than DeepConvLSTM. The classification time of testing instances (namely testing time) by Random Forest, DeepConvLSTM and Attention-RNN was 29.62 s, 9.82 s and 3.75 s, respectively. The test speed of Attention-RNN was 7.8 times that of Random Forest and 2.6 times that of DeepConvLSTM. The proposed Attention-RNN was more efficient than Random Forest and DeepConvLSTM. Although the test speed of Attention-RNN was slightly slower than that of CNN, the classification F1 value was greater than that of CNN. The above comparison results prove the beneficial effect of the proposed Attention-RNN. The proposed Attention-RNN had the largest F1 score in the ML task, the second F1 score in the GR task, and the second running speed. It achieved the optimal balance between F1 score and running efficiency.

**Figure 4:**
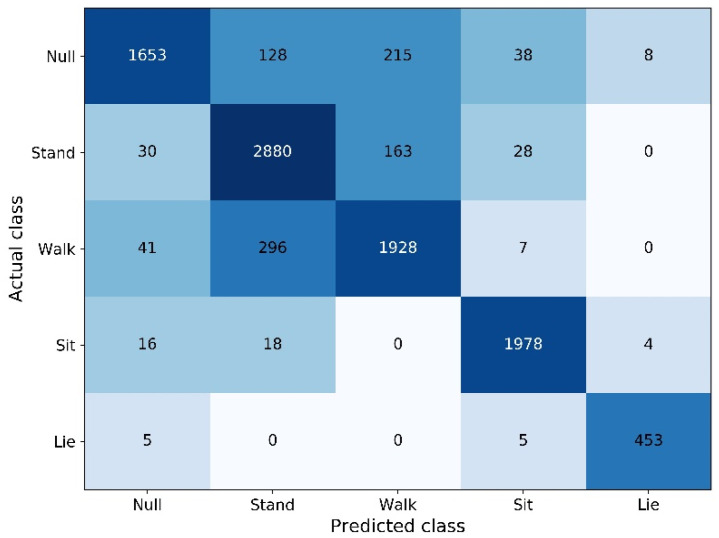
Confusion matrix of ML task.

The confusion matrix in Figure 4 shows the test results of Attention-RNN in the ML task. It can be seen from the figure that many Walk samples were misidentified as Stand and Null, and many Stand samples were misidentified as Walk and Null. Since the Walk samples were collected during daily indoor activities, the motion range was small. Therefore, Walk, Stand, and Null had certain similarities, and it was easy to identify them incorrectly.

**Figure 5:**
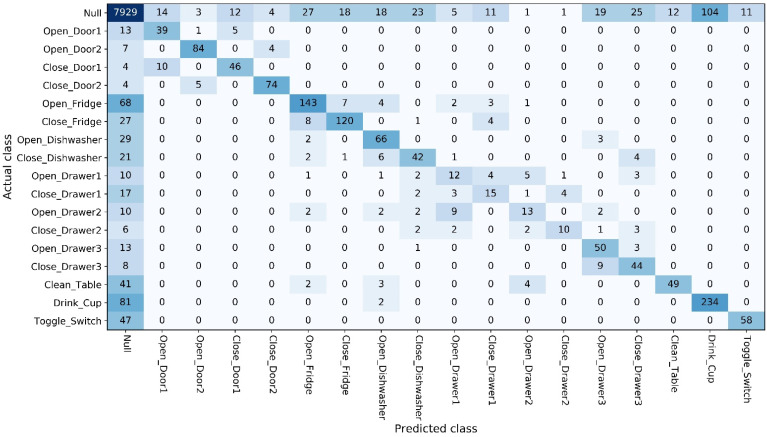
Confusion matrix of GR task.

The confusion matrix in Figure 5 shows the test results of Attention-RNN in the GR task. Most of the errors were related to the Null class. The main reason is that the classes of the dataset are extremely unbalanced, with Null classes accounting for 83.25% of the total samples.

The ablation experiments in Table 3 show the F1 score changes resulting from adding or removing different components of the Attention-RNN. The models of this set of experiments were all changed based on Attention-RNN. Model “A” removed the attention layer. Its F1 (ML) was 0.004 lower than Attention-RNN, and F1 (GR) was 0.008 lower than Attention-RNN. Model “B” removed the BN layer. Its F1 (ML) was 0.007 lower than Attention-RNN, and F1 (GR) was 0.005 lower than Attention-RNN. Models “D” and “E” changed the position of the BN layer, and their F1 scores were lower than the Attention-RNN. Models “I” and “J” changed the position of the Attention layer, and their F1 scores were not as good as Attention-RNN. Since Attention-RNN was only 0.01 orders higher than the F1 scores of the above models and the estimated F1 scores had uncertainty, it was unclear if it indicated an improvement. Models “C”, “F”, “G”, and “H” changed the number of BiLSTM layers. The Attention-RNN model with 2 BiLSTM layers had a larger F1 score than other models. Model “K” and “L” had two attention layers, and model “M” had three attention layers. The F1 scores of models “K”, “L” and “M” were all lower than Attention-RNN. The above results showed that increasing the number of attention layers or BiLSTM layers based on Attention-RNN did not improve the classification performance. In general, this set of experiments provided guidance for the establishment of the Attention-RNN.

A set of cross-validation experiments was implemented to verify the stability of Attention-RNN. First, the training set in Section 4.1 was randomly divided into two sub-training sets of the same size. Then, in the ML task, the two sub-training sets were used to train two models, M1 and M2, respectively. In the GR task, the two sub-training sets were used to train two models G1 and G2, respectively. Finally, the above four trained models were tested on the test set in Section 4.1. The test F1 scores of M1, M2, G1, and G2 were 0.886, 0.894, 0.894, and 0.895, respectively. The results show that even if half of the training set is used to train Attention-RNN, good classification results can be achieved. Besides, the difference between M1 and M2 and the difference between G1 and G2 were relatively small. Then, the stability of Attention-RNN had been verified.

#### 4.4.2. Experiments on Information Gain-Based Human Activity Model

**Figure 6:**
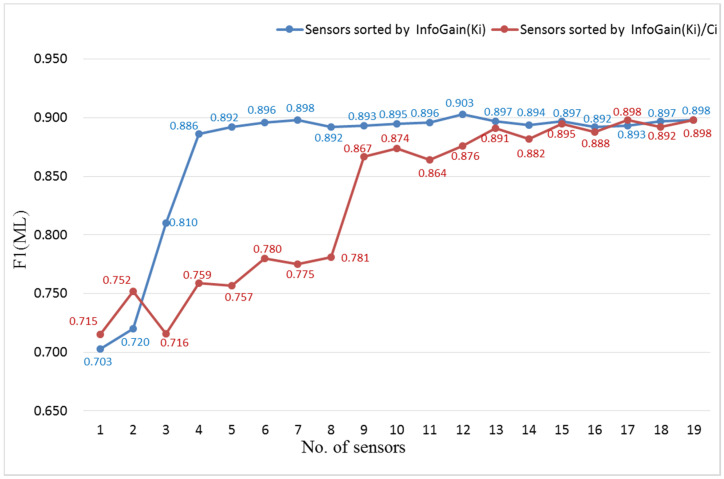
F1 scores for ML task with different numbers of sensors.

**Figure 7:**
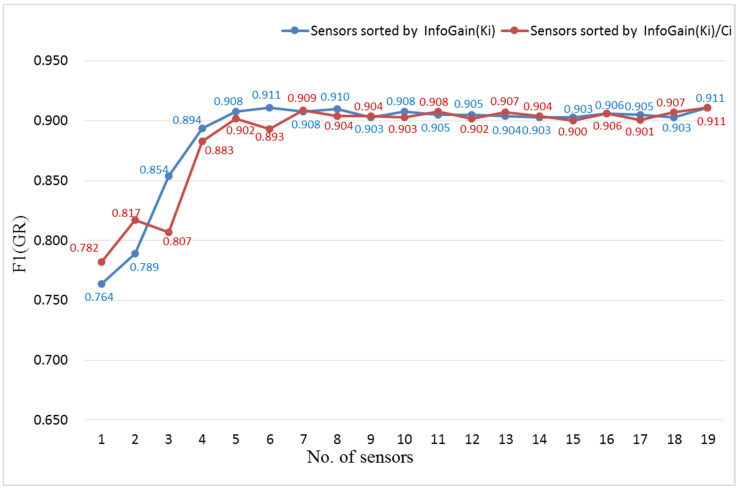
F1 scores for GR task with different numbers of sensors.

To verify the validity of the human activity model, another set of experiments was carried out as follows: First, the information gain of each sensor was calculated according to the Formulas (3)–(6). The training set (including the validation set) without sliding window processing was used to calculate the information gain. Each sensor channel was selected as a feature, so that the F in the equations referred to each sensor channel, and the v referred to the data of the sensor channel. Since there are multiple feature selection methods, it may lead to different feature selection criteria and feature rankings. This set of experiments can only verify the effect of the proposed feature selection method. For the ML task and GR task, the information gain of each sensor was shown in Table 4. Second, the top n (1, 2, 3, … 18, 19) information gain sensors’ data were used for training and testing Attention-RNN in turn, and the results are shown in Figure 6 and Figure 7.

F1 scores for ML tasks with different numbers of sensors are shown in Figure 6. For example, when the number of sensors is 2, 2 refers to the sensors with the top 2 information gain, namely L-SHOE and R-SHOE. The blue line in Figure 6 represents that the sensors are sorted by the sensor information gain $\mathrm{InfoGain}\left(K_{i}\right)$, which is the sum of the information gain over all channels of each sensor. The red line represents a set of comparative experimental results, and represents the sensors are sorted by $\mathrm{InfoGain}\left(K_{i}\right)/C_{i}$, which is the average of information gain over all channels of each sensor. In the experiments represented by the blue line, the F1 value continued to increase as the number of sensors increased from 1 to 7. When the number of sensors was 7, the F1 score reaches the same maximum value as 19 sensors. When the number of sensors was 12, the F1 score was 0.903, which reached the maximum and exceeded 0.898 of 19 sensors. In the comparative experiments represented by the red line, the F1 score fluctuated and rose as the number of sensors increased from 1 to 17. When the number of sensors was 17, the F1 score reached the same maximum value of 0.898 as with all 19 sensors. The experiments represented by the blue line required fewer sensors than the experiments represented by the red line to achieve the high-level F1 score. Therefore, top 12 sensors sorted by the sensor information gain $\mathrm{InfoGain}\left(K_{i}\right)$ can meet the requirements of ML task.

F1 scores for GR tasks with different numbers of sensors are shown in Figure 7. The blue and red lines in Figure 7 represent the experiments of two different sensor sorting methods, which are similar to Figure 6. In the experiments represented by the blue line, the F1 score steadily increased to the maximum value of 0.911 when the number of sensors gradually increased to 6. The F1 score of the experiment represented by the red line reached 9.09 when the number of sensors was 7, but it was smaller than that of the blue line with 6 sensors. Therefore, top 6 information gain sensors sorted by the sensor information gain $\mathrm{InfoGain}\left(K_{i}\right)$ are enough to meet the requirements of GR task, and there is no need to continue increasing the number of sensors.

**Figure 8:**
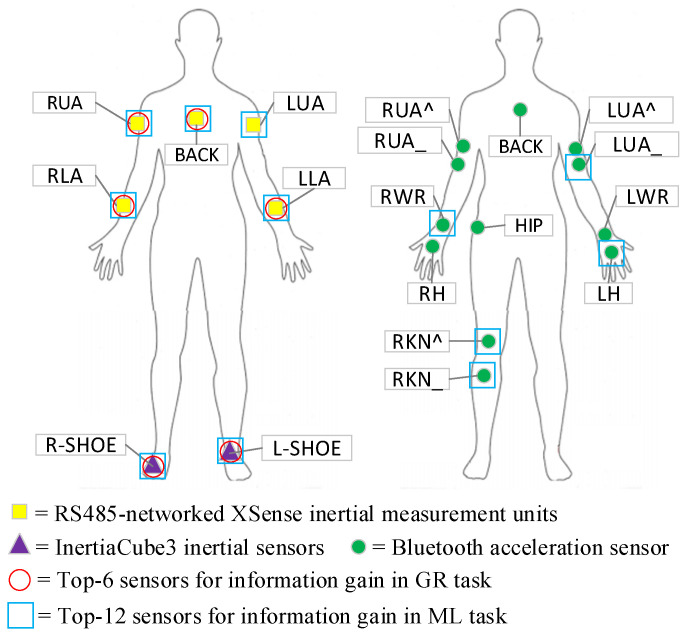
Top 6 information gain sensors in GR task and top 12 information gain sensors in ML task.

The red circle in Figure 8 marks the sensors with the top 6 information gain in the GR task, and the blue box marks the sensors with the top 12 information gain in the ML task. The sensors with top 6 information gain are mainly distributed on the arms and back, which are consistent with the characteristics of the upper limbs required to complete the GR task. Because completing the four activities in the ML task requires the cooperation of the upper and lower limbs, the top 12 information gain sensors that can achieve a good classification effect are distributed in the upper and lower limbs.

## 5. Conclusions

This paper proposed an information gain-based human activity model and an Attention-RNN for wearable sensor-based HAR. The experimental results on the UCI Opportunity Challenge dataset show that the proposed Attention-RNN has high accuracy and operating efficiency. The F1 score of the proposed Attention-RNN was 0.03 higher than the DeepConvLSTM in the 5-class ML task and 0.04 lower in the 18-class GR task. The test speed of the proposed Attention-RNN was 2.6 times that of DeepConvLSTM. At the same time, experiments prove that the proposed information gain-based human activity model provides a quantitative basis for the deployment of the sensors and fills the research gap in this field. The same classification effect can be achieved by using fewer sensors with high information gain, which can reduce the amount of calculation.

In the future, classification algorithms will be studied to further improve the classification effect. In addition, methods to solve the problem of data imbalance will also be explored. Finally, the stability of the overall control will be proved and its complete theorem will be put forward.

## Figures and Tables

**Table 1:** Composition of the dataset intercepted by the sliding window.

Task	Activity Name	# of Training Instances	# of Testing Instances
GR	Open_Door1	864	58
	Open_Door2	887	95
	Close_Door1	806	60
	Close_Door2	846	83
	Open_Fridge	921	228
	Close_Fridge	850	160
	Open_Dishwasher	666	100
	Close_Dishwasher	628	77
	Open_Drawer1	490	39
	Close_Drawer1	413	42
	Open_Drawer2	457	40
	Close_Drawer2	416	26
	Open_Drawer3	566	67
	Close_Drawer3	564	61
	Clean_Table	904	99
	Drink_Cup	3246	317
	Toggle_Switch	623	105
	Null	32348	8237
ML	Stand	19321	3101
	Walk	10875	2272
	Sit	7410	2016
	Lie	1209	463
	Null	7680	2042

**Table 2:** F1 comparison of different classification algorithms.

Method	F1 (ML Task)	F1 (GR Task)	Testing Time (S)
Random Forest [43]	0.870	0.900	29.62
CNN [44]	- ^1^	0.851	2.29
DeepConvLSTM [29]	0.895	0.915	9.82
Attention-RNN (ours)	0.898	0.911	3.75

^1^ “-” means there is no relevant data in the original paper.

**Table 3:** Experiments on different model structures.

Model	Structure	F1 (ML Task)	F1 (GR Task)
A	BN + 2BiLSTM + Dense	0.894	0.903
B	2BiLSTM + Attention + Dense	0.891	0.886
C	BN + 1BiLSTM + Attention + Dense	0.891	0.899
D	2BiLSTM + BN + Attention + Dense	0.893	0.903
E	2BiLSTM + Attention + BN + Dense	0.894	0.903
F	BN + 3BiLSTM + Attention + Dense	0.891	0.904
G	BN + 4BiLSTM + Attention + Dense	0.891	0.901
H	BN + 5BiLSTM + Attention + Dense	0.891	0.906
I	BN + Attention + 2BiLSTM + Dense	0.878	0.898
J	BN + BiLSTM + Attention + BiLSTM + Dense	0.892	0.891
K	BN + BiLSTM + Attention + BiLSTM + Attention + Dense	0.890	0.901
L	BN + Attention + BiLSTM + Attention + BiLSTM + Dense	0.881	0.899
M	BN + Attention + BiLSTM + Attention + BiLSTM + Attention + Dense	0.857	0.898
Attention-RNN	BN + 2BiLSTM + Attention + Dense	0.898	0.911

**Table 4:** Information gain and ranking of each sensor.

Sensor Name	Channels	$InfoGain\left(K_{i}\right)\;\mathrm{of}\;\mathrm{ML}\;\mathrm{Task}\;(\mathrm{Ranking})$	$InfoGain\left(K_{i}\right)\;\mathrm{of}\;\mathrm{GR}\;\mathrm{Task}\;(\mathrm{Ranking})$
RKN^	1–3	1.797 (8)	0.558 (15)
HIP	4–6	0.840 (18)	0.471 (19)
LUA^	7–9	1.092 (13)	0.615 (12)
RUA_	10–12	0.927 (16)	0.600 (14)
LH	13–15	1.617 (9)	0.972 (9)
BACK (Acc)	16–18	0.861 (17)	0.618 (11)
RKN_	19–21	1.332 (10)	0.603 (13)
RWR	22–24	1.308 (11)	1.464 (8)
RUA^	25–27	0.822 (19)	0.474 (18)
LUA_	28–30	1.119 (12)	0.510 (16)
LWR	31–33	1.011 (14)	0.492 (17)
RH	34–36	0.963 (15)	0.741 (10)
BACK (IMU)	37–45	2.817 (3)	2.088 (3)
RUA	46–54	2.610 (6)	1.890 (6)
RLA	55–63	2.241 (7)	1.971 (4)
LUA	64–72	2.664 (5)	1.818 (7)
LLA	73–81	2.772 (4)	1.899 (5)
L-SHOE	82–97	4.832 (1)	2.400 (2)
R-SHOE	98–113	4.784 (2)	2.448 (1)

## Data Availability

Publicly available dataset was analyzed in this study. This dataset can be found here: https://archive.ics.uci.edu/ml/datasets/OPPORTUNITY+Activity+Recognition, accessed on 2 December 2021.
